# Changes in pornography use and sexual behaviour during lockdown

**DOI:** 10.1192/j.eurpsy.2021.94

**Published:** 2021-08-13

**Authors:** M. Koós, B. Bőthe, O. Király, B. Paksi, Z. Demetrovics

**Affiliations:** Institute Of Psychology, ELTE Eötvös Loránd University, Budapest, Hungary

**Keywords:** Sexual Behavior, sexual life satisfaction, quarantine, sexual life frequency

## Abstract

COVID-19 pandemic and the related restrictions had a significant impact on the living and working conditions as well as the everyday behavior and mental health condition. Aim of the current analysis was to examine the impact of the input-deprived circumstances on the sexual life characteristics. An online survey carried out after a few weeks of the first nation-wide lockdown was enacted in Hungary. 1,755 persons participated in the first wave (50.4% males). Relationship and sexual life satisfaction, sexual intercourse and masturbation frequency were assessed with additional single-item questions about the subjective change (5-pont Likert scale; 1=“decreased significantly”; 5=“increased significantly) in these characetristics since the epidemiological restrictions had been introduced. Furthermore, several potential protective and risk factors were measured (depressive symptoms, perceived stress, loneliness, general well being, intolerance of uncertainty, sensation seeking, and COVID-19 related health anxiety). Linear regression models were calculated to assess which of the former variables predict the subjective changes of one’s sexual life. The analyses resulted in weak standardized coefficients. The subjective change in relationship satisfaction (mean 3.20, SD 0.94) and sexual satisfaction (mean 2.82, SD 0.73) were predicted positively by general well-being (β=.11–0.25, p<.01), and negatively by loneliness (β=-.14-0.19, p<0.01). Loneliness predicted negatively the subjective change in sexual intercourses (mean 2.75, SD 0.89) and masturbation frequency (mean 2.89, SD 0.84) (β=- .10–.12, p<.01), while sensation seeking had no effect (β=-.09, p<.01). The COVID-19 related health anxiety predicted negatively only the change in sexual life frequency (β=-.07, p<.05). The explained variances were rather small (1.7%-11.8%).

**Disclosure:**

No significant relationships.

